# Transcriptome profiles reveal gene regulation of ginger flowering induced by photoperiod and light quality

**DOI:** 10.1186/s40529-023-00388-7

**Published:** 2023-05-27

**Authors:** Qinyu Deng, Yangtao Zhang, Kang Liu, Guo Zheng, Longyan Gao, Zhexin Li, Mengjun Huang, Yusong Jiang

**Affiliations:** 1 College of Biology and Food Engineering, Chongqing Three Gorges University, Chongqing, 404000 China; 2 Research Institute for Special Plants, Chongqing University of Arts and Sciences, Chongqing, 402160 China; 3 College of Resources and Environment, Southwest University, Chongqing, 400715 China

**Keywords:** Flowering mechanism, Ginger, Long photoperiod, Red light treatment, Transcriptome sequencing

## Abstract

**Background:**

Under natural conditions, ginger (*Zingiber officinale* Rosc.) rarely blossom and has seed, which limits new variety breeding of ginger and industry development. In this study, the effects of different photoperiods and light quality on flowering induction in ginger were performed, followed by gene expression analysis of flower buds differentiation under induced treatment using RNA-seq technology.

**Results:**

First, both red light and long light condition (18 h light/6 h dark) could effectively induce differentiation of flower buds in ginger. Second, a total of 3395 differentially expressed genes were identified from several different comparisons, among which nine genes, including *CDF1*, *COP1*, *GHD7*, *RAV2-like*, *CO*, *FT*, *SOC1*, *AP1* and *LFY,* were identified to be associated with flowering in induced flower buds and natural leaf buds. Aside from four down-regulated genes (*CDF1*, *COP1*, *GHD7* and *RAV2-like*), other five genes were all up-regulated expression. These differentially expressed genes were mainly classified into 2604 GO categories, which were further enriched into 120 KEGG metabolic pathways. Third, expression change of flowering-related genes in ginger indicated that the induction may negatively regulated expression of *CDF1*, *COP1*, *GHD7* and *RAV2-like*, and subsequently positively regulated expression of *CO*, *FT*, *SOC1*, *LFY* and *AP1*, which finally led to ginger flowering. In addition, the RNA-seq results were verified by qRT-PCR analysis of 18 randomly selected genes, which further demonstrated the reliability of transcriptome analysis.

**Conclusion:**

This study revealed the ginger flowering mechanism induced by light treatment and provided abundant gene information, which contribute to the development of hybrid breeding of ginger.

**Supplementary Information:**

The online version contains supplementary material available at 10.1186/s40529-023-00388-7.

## Introduction

Ginger (*Zingiber officinale* Roscoe) is a perennial monocotyledon from the Zingiberaceae family, which is not only an important condiment but also one of the most commonly used Chinese medicines (Miri 2020). Ginger is used worldwide as a flavoring agent in the bread, food, beverage, and bakery. In addition, ginger is commonly used in the pharmaceutical industry as a herbal medicine for treating vomiting, colds, fever and even cancer (Park et al. 2014; Thomson et al. 2014). The flower buds of ginger may developed directly from the rhizome, or it may originated from leaf buds. A pseudo stem could generated and elongated from 3–5 leaves after leaf buds development, and flower buds subsequently emerged at the middle of pseudo stem (Melati et al. 2015). During the growth of flower buds, it gradually changed from slender to round, and formed spikes. Then, flower emerged from each spike, one flower per bract up to three flowers (Melati et al. 2015). However, ginger rarely flowers or sets seed under natural conditions, and it mainly relies on rhizomes for asexual propagation in the cultivation process (Malamug et al. 1991; Sajeev et al. 2011; Melati et al. 2015). Studies have shown the effect of photoperiod and light intensity on ginger flowering (Adaniya et al. 1989; Melati et al. 2015), but floral regulatory genes have not been revealed. Therefore, it is difficult to obtain new cultivars through hybrid breeding, which greatly limits the development of ginger industry.

Environmental factors (such as light and temperature) and endogenous factors (such as plant hormones and age) could regulate flowering time in plants. Light quality and photoperiod control photomorphogenesis, which play an important role in flowering of various plants (Zhang et al. 2022; Sidhu et al. 2021). Plants could sense photoperiod variation of light with different wavelengths through photoreceptor proteins, such as phytochrome or cryptochrome, which would be signals to initiate flower (Weller and Kendrick 2008). For example, long light (16 h/8 h and 24 h/0 h) promoted flowering of *Lysimachia mauritiana* Lam at low temperature of 5 °C (Im et al. 2020). A significant decrease of flowering time was also observed for *Arabidopsis thaliana* mutants when constant fluorescent illumination was supplemented with irradiation enriched in red and far red spectrum (Somerville 1990). In photoperiod pathway, *CDF1* and *GI* sense changes of circadian rhythms and transmit signals to *CO* gene to obtain more CO protein, high CO protein levels then activate the *FT* gene expression, which in turn promotes the expression of *LFY* and *AP1* genes, and finally induce flowering (Valverde et al. 2004). *CDF1* in *A. thaliana* could cause flowering delay by repressing the transcription of *CO* and *FT* genes (Sawa et al. 2007). In addition, *COP1* inhibited flowering by degrading CO protein in *A. thaliana* (Liu et al. 2008).

Transcriptome analysis could accurately obtain transcriptional expression of all genes of studied plant using RNA-seq sequencing, thus provides possibility of important gene mining from obtained data (Sangwan et al. 2013). For example, flowering related genes were identified in transcriptome analysis of strawberry leaves, which reveals flowering regulation mechanism of strawberry under blue light treatment (Ye et al. 2021). Therefore, the identification of genes related to flowering in ginger transcriptome data would also contribute to elucidate the mechanism of induced ginger flowering. In this study, the optimal treatment of different photoperiod and light quality was screened for ginger. Then, an illumina high-throughput sequencing was used to analyze the transcriptional expression during flower bud formation. This study aimed to explore the mechanism for flowering in ginger and provide new insight for future breeding projects.

## Materials and methods

### Plant material and treatments

The ginger tissue materials involved in experiment were taken from tissue culture seedlings of *Zingiber officinale* Roscoe cv. Southwest in plant germplasm resource nursery of Chongqing University of Arts and Sciences (Chongqing China, N25° 56′ N, 105° 38′ E). Ginger seedlings were planted in a germplasm resource nursery and grow under green house conditions (temperature: 25 °C, humidity: 60%, light intensity: 200 μEm^−2^ s^−1^, photoperiod: 14 h light/10 h dark) to resume growth to three-forked shape stage.

For flowering induction, these ginger plants were grown in a greenhouse treated with the following four photoperiods: T1 (14 h light/10 h dark), T2 (16 h light/8 h dark), T3 (18 h light/6 h dark), and T4 (20 h light/4 h dark). In total, there were 10 pots for each treatment with two plants per pot. Each experimental condition was independently repeated three times. The flowering period of ginger is about 7 months after sowing, which indicates the physiological differentiation of ginger flower buds takes place at 40–60 days after three-forked shape stage (Kumari et al. 2020). Therefore, the number of ginger flower buds in each treatment was counted at 60 days and 70 days after three-forked shape stage to obtain the optimal photoperiod for flowering induction. The formula of flower bud differentiation rate is as follows:

1 $${\text{Flower bud differentiation rate }}\left( \% \right) = \left[ {{\text{number of flower buds}}/\left( {{\text{number of flower buds}} + {\text{number of leaf buds}}} \right)} \right] \times {1}00\% .$$

In light quality, ginger plants were cultivated under different colored lights by treated with four different films: red film (R), blue film (B), green film (G) and white film (CK). The photoperiod was set to 18 h light/6 h dark based on previous result. The flower buds were also counted, and the optimal flower-inducing light quality was screened. In total, there were 10 pots per treatment with two plants in each pot, and three replicates were conducted.

### Samples collection

Based on previous screened results, ginger tissue culture seedlings were induced to flower with the optimal photoperiod and light quality. Potential flower buds (FI), leaf buds (LI) and leaf buds (LN) under natural conditions (white light and 14 h light/10 h dark) of ginger plants were simultaneously collected at physiological differentiation of ginger flower buds (after 50 days of treatment). These samples were rapidly frozen in liquid nitrogen and stored at – 80 °C. Three independent biological replicates were performed for each treatment.

### RNA isolation and library preparation

RNA was extracted using the TRIzol method (Invitrogen, CA, USA) and treated with RNase-free DNase I (Takara, Kusatsu, Japan). RNA was quantified using Agilent 2100 Bioanalyzer (Agilent Technologies, CA, USA), then its quality and integrity were assessed using NanoDrop spectrophotometer (Thermo Scientific, DE, USA). Sequencing libraries were constructed using NEBNext^®^ Ultra™ RNA Library Prep Kit for Illumina^®^ (NEB, USA). Finally, the library were sequenced on the Illumina Novaseq 6000 platform by the Beijing Allwegene Technology Company Limited (Beijing, China) and 150 bp paired-end reads were generated.

### Transcriptome assembly and functional annotation of transcripts

Raw data (raw reads) with fastq format were processed through in-house perl scripts. To obtain clean data, the reads containing ploy-N and low quality reads were removed from raw data. Then, these clean reads were mapped to reference genome sequence using STAR (Dobin et al. 2012). To obtain unigene library, sequence assembly was performed using Trinity 2.2.0 software (Haas et al. 2013). The transcripts less than 300 bp were discarded and the longest transcript in each cluster was taken as unigene. Unigenes were blasted with non-redundant protein sequences in Non-redundant protein sequence database (NR), gene ontology (GO), Kyoto encyclopedia of genes and genomes (KEGG), search tool for the retrieval of interacting genes/proteins (STRING) databases to obtain annotated information using blastp software (Camacho et al. 2009). E-value distribution was calculated and plotted according to NR library comparison annotation, and the screening criterion was set as E value < 1.0 × e^−5^.

### Identification of differentially expressed genes

Unigenes were mapped to raw reads using Bowtie2 software (Langmead and Salzberg 2012). The expression level of single gene was calculated and normalized using Expectation Maximization in RSEM, and were estimated using the most commonly used FPKM (Li and Dewey 2011). Differentially expressed unigenes (DEGs) with significance in different groups were identified using the edgeR package in R language (Smyth 2010), with threshold value of |log2 (Fold Change)|≥ 1 and q-value ≤ 0.05. GO and KEGG enrichment analysis were performed using hypergeometric distribution in R language (Alexa et al. 2006).

### qRT-PCR validation

Eighteen representative DEGs were randomly selected from the comparison groups (FI vs. LN, FI vs. LI and LI vs. LN) for qRT-PCR validation. The *actin1* gene was used as internal reference, and all primer sequences were designed using Primer 5.0. Amplification reactions were conducted in a total volume of 20 µl. Cycling parameters were set as follows: 95 °C for 30 s, then 40 cycles of 95 °C for 5 s and 60 °C for 30 s. Gene expression was calculated using 2^−∆∆Ct^ method, and all qRT-PCR reactions were repeated three times.

## Results

### Effects of photoperiod and light quality on flowering in ginger

**Fig. 1:**
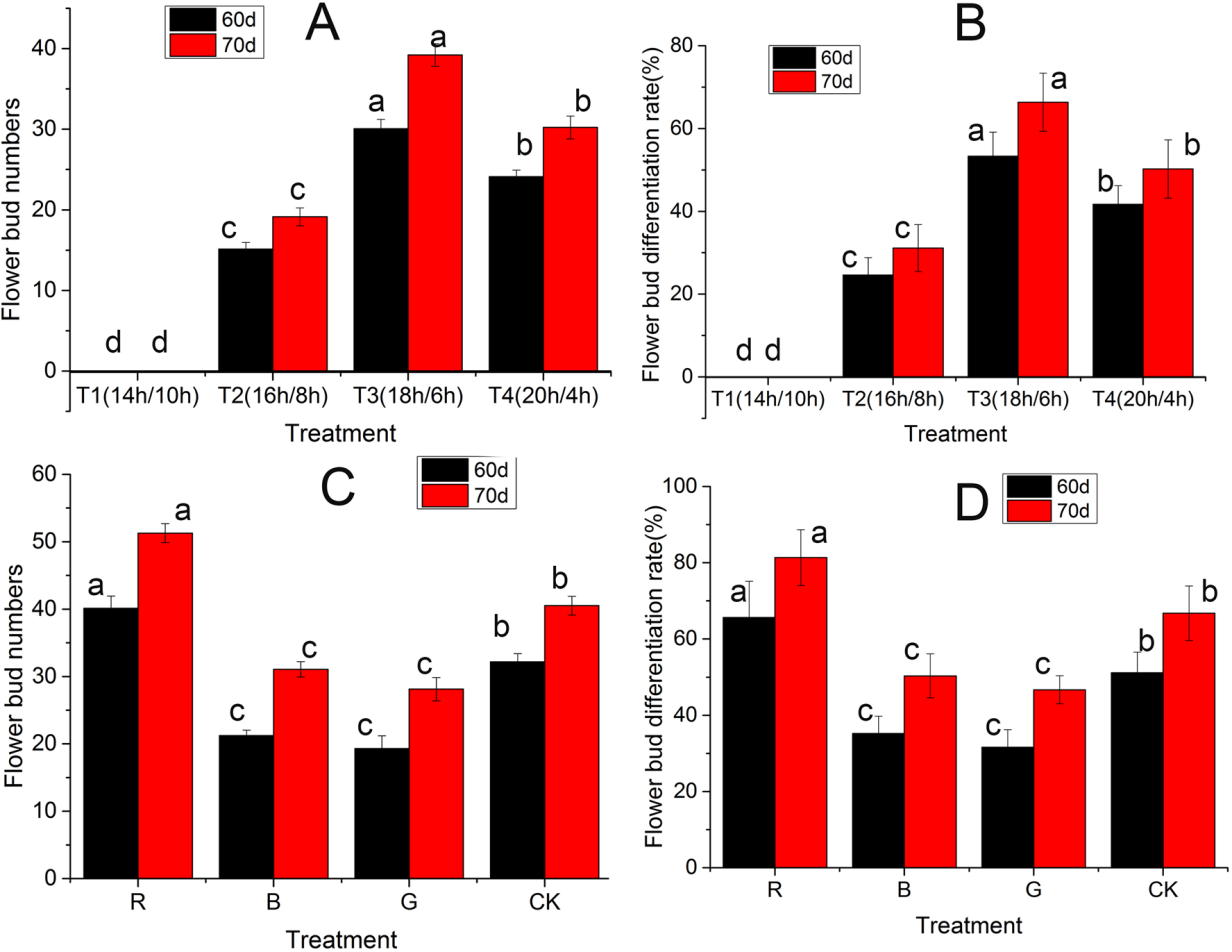
The effects of different photoperiod and light quality on ginger flowering. Treatments marked with different letters at a given statistics date are significantly different at P < 0.05.according to Duncan’s test. **A** The effects of different photoperiod on flower bud numbers; **B** the effects of different photoperiod on flower bud differentiation; **C** the effects of different light quality on flower bud numbers; **D** the effects of different light quality on flower bud differentiation

In T2, T3 and T4 photoperiod groups, the flower bud differentiation rates of ginger were 24.62%, 53.33% and 41.67%, respectively. None of flower buds differentiation was observed in T1 photoperiod treatment during whole experiment process. After 70 days, T3 treatment group had the most flower buds number with the most significant increase, from 30.06 on 60 days to 39.21 on 70 days (Fig. 1A, B). These findings indicated that long light condition (18 h/6 h) was beneficial to induced differentiation of ginger flower buds. The light quality results showed that flower bud differentiation rates of ginger under R (red film) treatment were higher than that in control group (white film), with 40.12 and 51.26 flower buds on 60 days and 70 days under R-treated treatment, respectively (Fig. 1C, D). There was no significant difference in flower bud differentiation rate between B (blue film) and G (green film) treatments, but they was significantly lower than that of control group. These results indicated that red light could better trigger flowering in ginger.

### Statistics of transcriptome sequencing data

RNA-seq was performed for FI (potential flower buds under induced treatment), LI (leaf buds under induced treatment), and LN (leaf buds under natural condition) based on the HiSeq 2500 (Illumina) platform. After downloading raw data, these reads containing adapter and ploy-N and low quality reads were then removed, 59.02 Gb clean data were subsequently obtained. The clean reads of each sample were distributed in range of 40–51 million and more than 90% of reads were mapped in reference sequences (Table 1). The distribution of Q30 bases exceeded 90%, and GC contents ranged from 47.64 to 49.57%. These filtered reads were assembled using Trinity software (Haas et al. 2013), and 33,612 unigenes were obtained with an N50 value of 1123 bp and 40,384,342,800 bp transcriptome data. Functional annotation of proteins was performed using BLASTP based on GO, KEGG, STRING and NR non-redundant protein databases, and a total of 29,985 unigene sequences was annotated (89.21%) (Additional file 1: Table S1).

**Table 1:** Statistics of ginger transcriptome data

Sample	Raw reads	Clean reads	Tatal Mapped	Multiple mapped	Q30	GC content
FI1 FI2 FI3	44,582,118 42,945,294 43,894,564	43,627,860 41,428,602 42,428,512	40,657,844 (93.19%) 38,887,172(93.87%) 39,982,225 (94.23%)	1,175,032 (2.69%) 1,230,258 (2.97%) 1,182,543 (2.87%)	94.34% 94.56% 94.88%	48.23% 49.57% 48.96%
LI1 LI2 LI3	42,635,092 42,281,372 42,123,265	41,560,670 40,330,888 40,930,555	38,993,816 (93.82%) 37,712,828 (93.51%) 37,899,887 (92.60%)	1,124,250 (2.71%) 1,171,920 (2.91%) 1,162,547 (2.85%)	94.37% 94.61% 94.12%	48.58% 47.64% 48.21%
LN1 LN2 LN3	51,254,400 45,530,676 47,165,454	50,189,780 44,569,834 46,655,888	47,096,494 (93.84%) 41,882,894 (93.97%) 42,255,885 (90.56%)	1,369,568 (2.73%) 1,205,616 (2.71%) 1,269,853 (2.72%)	94.59% 94.82% 94.49%	48.03% 48.97% 49.25%

FI1–FI3: potential flower bud of ginger under induced by photoperiod and light quality; LI1–LI3: induction treatment of ginger leaf bud; LN1–LN3: leaf bud of ginger under natural condition

### Differentially expressed genes analysis

**Fig. 2:**
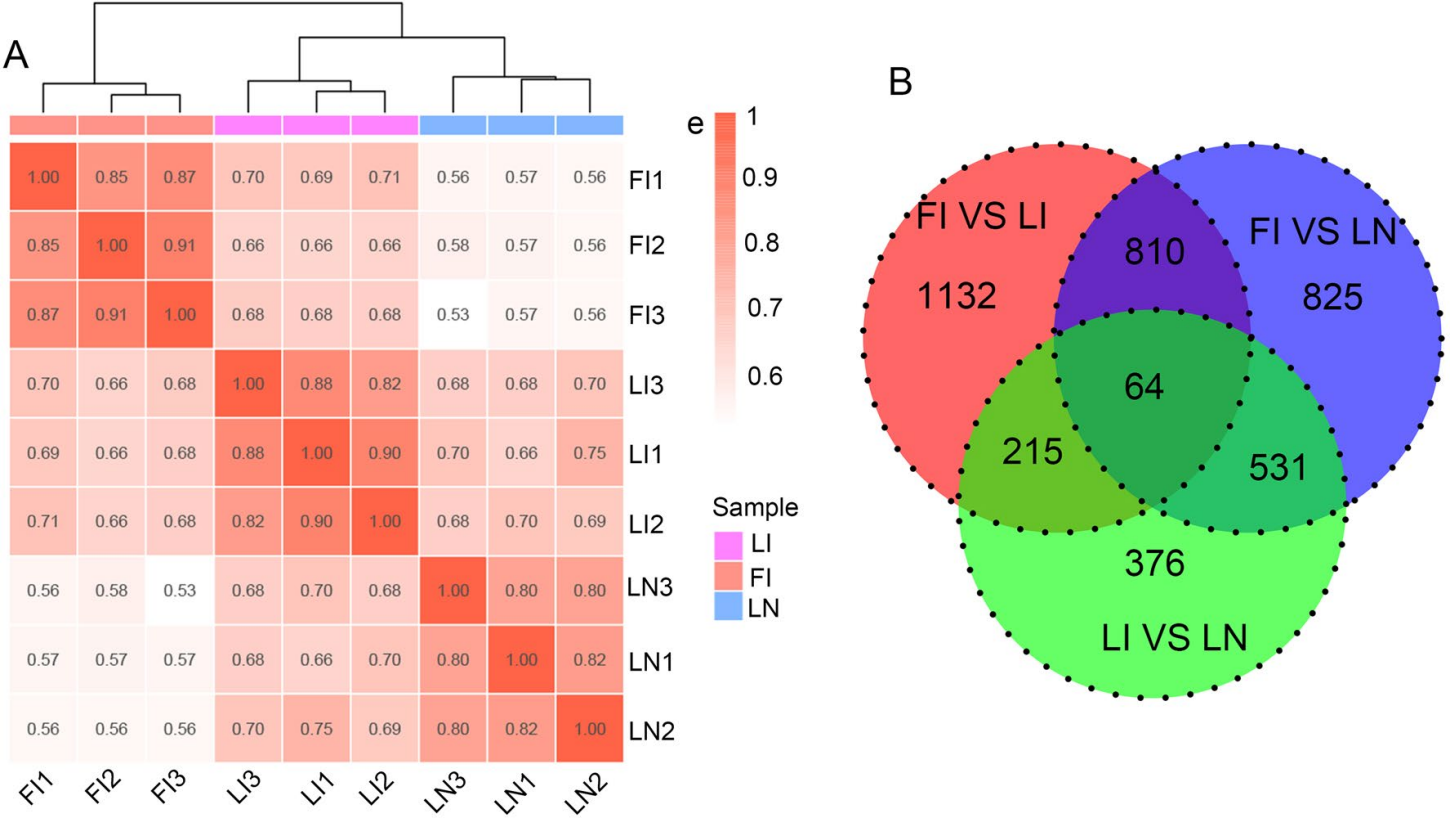
Differentially expressed unigenes (DEGs) of gingers among sample comparisons (FI: potential flower bud of ginger under induced by photoperiod and light quality; LI: induction treatment of ginger leaf bud; LN: leaf bud of ginger under natural condition). **A** Correlation analysis of sample groups; the number in the grid represents the square of Pearson correlation coefficient (R^2^) and the higher R^2^ represents the better correlation between samples. **B** Venn diagrams of DEGs between sample pairs

To clarify the induction mechanism of ginger flowering, a transcriptome analysis was performed for three samples of FI, LI and LN. Pearson correlation analysis on single-gene expression showed high correlation existed in three biological replicate samples, indicating a high quality and robust sequencing library (Fig. 2A). In comparison of FI and LN, a total of 2230 DEGs were identified (|log2 (Fold Change)|≥ 1 and q-value ≤ 0.05), with 1134 up-regulated genes and 1096 down-regulated genes. In comparison of tissues in LI and LN, a total of 1186 DEGs were identified, with 549 up-regulated genes and 637 down-regulated genes. There were 2221 DEGs identified in comparison between FI and LN tissues, including 1103 up-regulated genes and 1118 down-regulated genes. Besides, there were common 64 DEGs among all three comparisons (Fig. 2B).

To verify the accuracy of quantitative and differential expression, 18 DEGs were randomly selected, and primer sequences were designed for real-time quantitative PCR (qRT-PCR). The assays displayed a same trend of gene expression in qRT-PCR and RNA-Seq with a correlation coefficient of 0.85, which indicated that there was a good consistency between RNA-sequencing and qRT-PCR (Additional file 2: Fig. S1).

### Annotation and enrichment analysis of DEGs

GO annotation could help us understand the function of identified DEGs. The results revealed that 1699 DEGs identified in FI vs. LI were classified as biological process, cellular component and molecular function in GO database (Additional file 3: Fig. S2). There were 60 terms that were significantly enriched in GO database, which mainly involved with DNA-binding transcription factor activity, terpene synthase activity and transcription regulator activity. In LI vs. LN, 892 DEGs were annotated to the three types, of which 21 terms were significantly enriched in photosystem II oxygen evolving complex, oxidoreductase activity and photosystem I (Additional file 4: Fig. S3). The 1706 DEGs of FI vs. LN were annotated to biological processes, cellular components and molecular functions in GO database (Additional file 5: Fig. S4), of which 59 terms with significantly enriched genes were mainly protein phosphorylation, adenyl ribonucleotide binding and and thylakoid membrane.

**Fig. 3:**
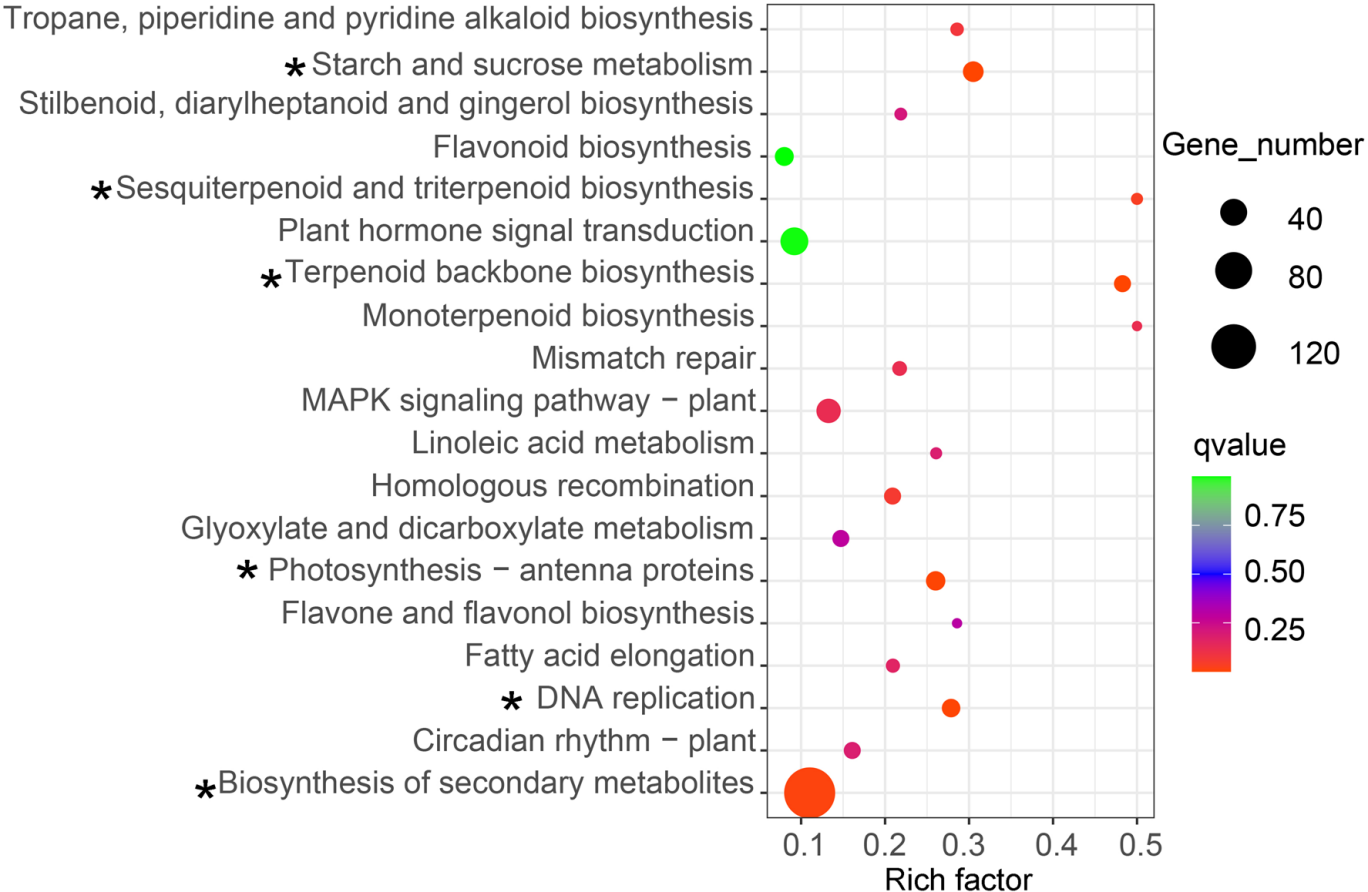
KEGG pathway enrichment of DEGs in FI vs. LI. Rich factor refers to the ratio of the number of differentially expressed genes enriched in the pathway to the number of annotation genes. Q value (range 0–1) is P value after correction by multiple hypothesis testing, and the closer the value of Q value is to 0, the more significant the enrichment is. Asterisks indicate significantly enriched pathways. (FI: potential flower bud of ginger under induced by photoperiod and light quality; LI: induction treatment of ginger leaf bud; LN: leaf bud of ginger under natural condition)

**Fig. 4:**
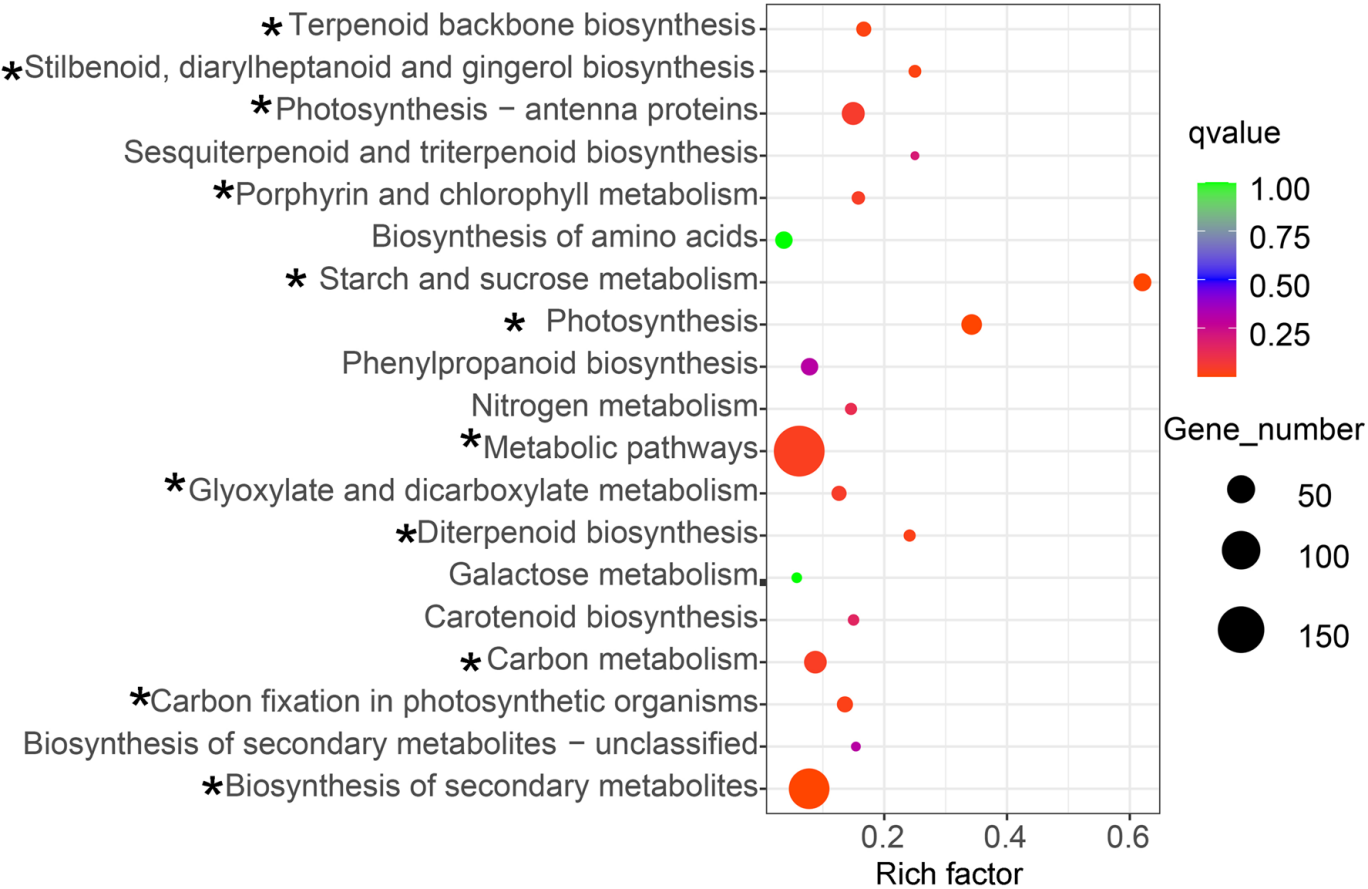
KEGG pathway enrichment of DEGs in LI vs. LN. Rich factor refers to the ratio of the number of differentially expressed genes enriched in the pathway to the number of annotation genes. Q value (range 0–1) is P value after correction by multiple hypothesis testing, and the closer the value of Q value is to 0, the more significant the enrichment is. Asterisks indicate significantly enriched pathways. (FI: potential flower bud of ginger under induced by photoperiod and light quality; LI: induction treatment of ginger leaf bud; LN: leaf bud of ginger under natural condition)

**Fig. 5:**
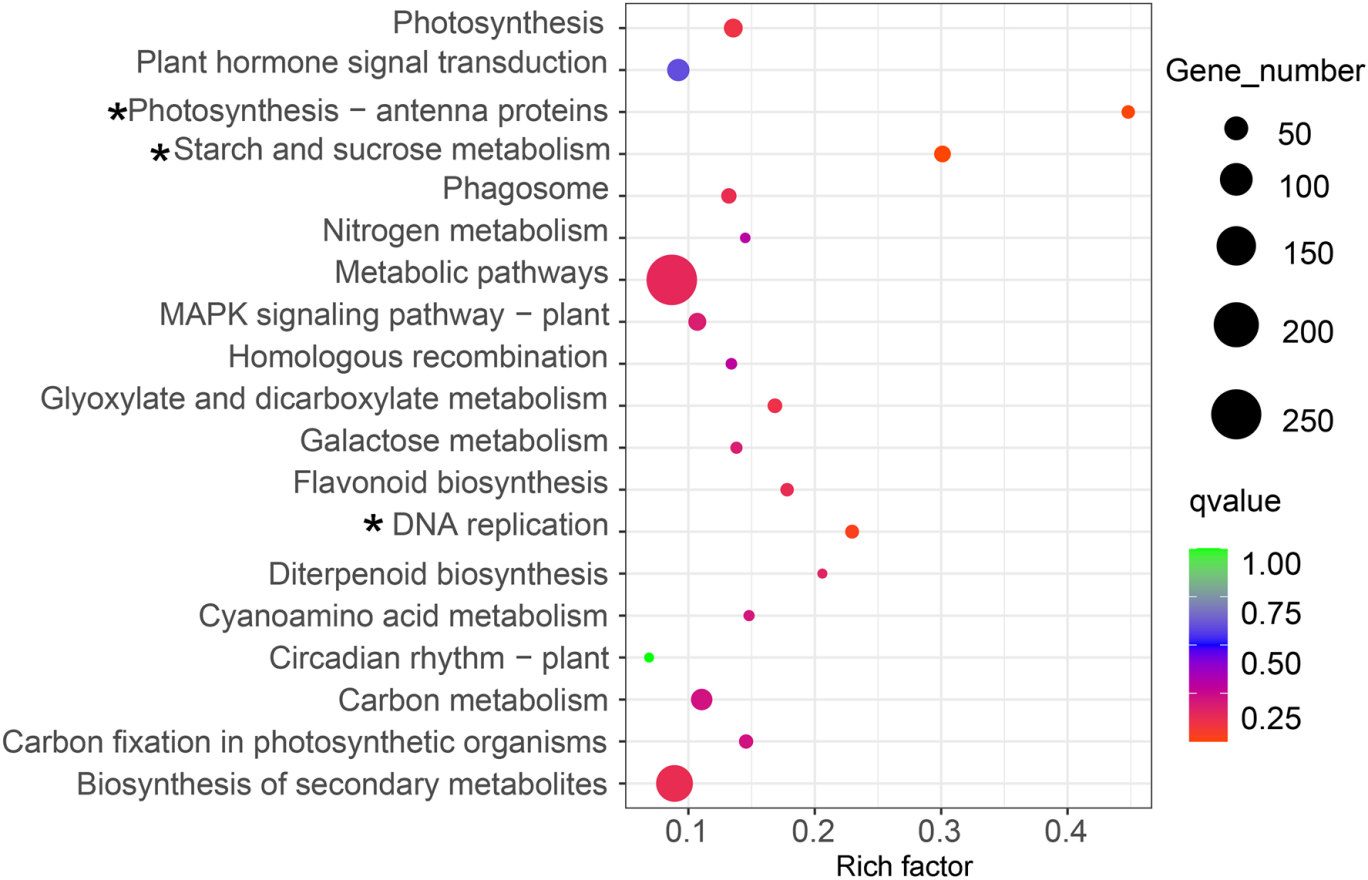
KEGG pathway enrichment of DEGs in FI vs. LN. Rich factor refers to the ratio of the number of differentially expressed genes enriched in the pathway to the number of annotation genes. Q value (range 0–1) is P value after correction by multiple hypothesis testing, and the closer the value of Q value is to 0, the more significant the enrichment is. Asterisks indicate significantly enriched pathways. (FI: potential flower bud of ginger under induced by photoperiod and light quality; LI: induction treatment of ginger leaf bud; LN: leaf bud of ginger under natural condition)

The hypergeometric test found that a total of 2230 DEGs were enriched in 120 KEGG pathways, and Figs. 3, 4 and 5 showed the top 19 pathways with the smallest Q values for each group. Among these pathways, only plant hormone signal transduction pathway and starch and sucrose metabolism pathway were found to be associated with flowering (Campos et al 2017; Wahl et al. 2013). In FI vs. LI, 43 DEGs were enriched in plant hormone signal transduction pathway, of which 17 genes were up-regulated and 26 genes were down-regulated. Meanwhile, 18 DEGs were enriched in starch and sucrose metabolism pathway, of which 8 genes were up-regulated and 10 genes were down-regulated (Fig. 3). There were 17 DEGs enriched in plant hormone signal transduction pathway including 12 up-regulated genes and 5 down-regulated genes in LI vs. LN. Eighteen DEGs were enriched in starch and sucrose metabolism pathway with 11 up-regulated genes and 7 down-regulated genes (Fig. 4). In FI vs. LN, 43 DEGs were enriched in plant hormone signal transduction pathway with 29 up-regulated genes and 14 down-regulated genes. Twenty nine DEGs were enriched in starch and sucrose metabolism pathway with 15 up-regulated genes and 14 down-regulated genes (Fig. 5).

### Screening of genes related to flowering in ginger

**Fig. 6:**
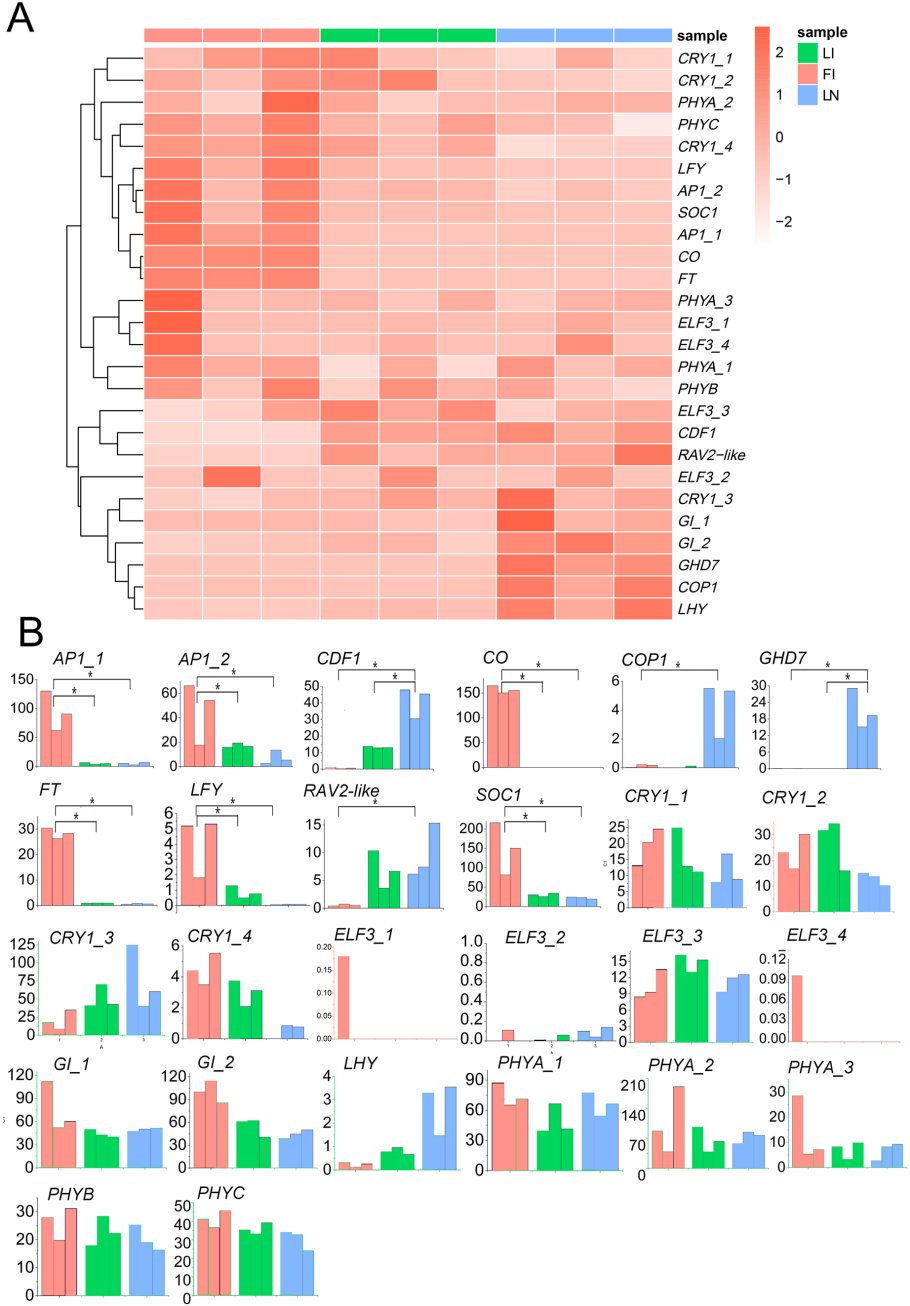
The expression profiles of flowering-related DEGs (FI: potential flower bud of ginger under induced by photoperiod and light quality; LI: induction treatment of ginger leaf bud; LN: leaf bud of ginger under natural condition). **A** A heatmap of flowering-related genes. **B** The FPKM value of flowering-related genes; the asterisk indicates that unigene is significantly differentially expressed in the comparison and each bar represents one biological replicate; FI, LI, and LN are indicated by red, green, and blue, respectively

Plants mainly sense and conduct signal transduction through various photoreceptors, and then regulate blossom (Lu et al. 2015). In this study, to find the relevant DEGs that regulate ginger flowering under different light qualities and photoperiods treatment, the transcriptome expression patterns of FI vs. LN were compared. Twenty six homologous genes in ginger were obtained through sequence alignment of ginger transcripts to coding sequence of *A. thaliana* and *Oryza sativa* genome (Komeda 2004; Izawa et al 2003). Among them, 10 genes related to flowering regulation were identified with significant differences which included *CDF1*, *COP1*, *GHD7*, *RAV*, *CO*, *FT*, *SOC1*, *AP1_1*, *AP1_2* and *LFY* (Fig. 6)*.* In FI vs. LN, four genes located in upsteam of flowering regulation were down-regulated expression while six genes lied in downsteam were up-regulated expression (Fig. 6). The annotation of these DEGs was shown in Additional file 6: Table S2. Compared to the control, fragments per kilobase of transcript per million mapped reads (FPKM) values of *CDF1*, *COP1*, *GHD7* and *RAV* in FI were decreased from 38.86, 3.76, 29.12 and 8.75 to 0.69, 0.12, 0.02 and 0.55, respectively. The expression levels of them were significantly decreased by 56.32, 31.33, 1456 and 15.9 fold, respectively. However, the expression of *CO*, *FT* and *SOC1* were all up-regulated with the FPKM values of 151.31/0.03, 28.34/0.62 and 141.25/20.36 in FI and LN and increased by 5043.67, 45.71 and 6.85 times, respectively (Fig. 6). The *AP1* genes at two different sites (*AP1_1* and *AP1_2*) and *LFY* genes were also up-regulated, with FPKM values of 96.31/13.65 (*AP1_1*), 41.23/8.33 (*AP1_2*) and 3.95/0.06 (*LFY*) in FI and LN (Fig. 6).

## Discussion

Alteration in photoperiods and light quality significantly affects flowering process (Baloch et al. 2009; Magar et al. 2018). Plants, such as Moss Rose, Pansy, Snapdragon, Petunia and Annual Verbena, had the minimum time to flower when grow under 17 h photoperiod while it became longer as photoperiod decreased to 8 h (Baloch et al. 2010). In cranberry, the number of flowers under red light was significantly more than that in white and far-red light (Zhou and Singh 2002). In our study, the flower bud differentiation rate of ginger had the highest value under red light and long light (18 h/6 h) treatments compared other groups, which was consistent with previous reports (Baloch et al. 2010; Zhou and Singh 2002). Therefore, further elucidation of flowering mechanism in response to light induction plays an important role in new varieties breeding of ginger. Besides, transcriptome analysis of induced potential flower buds and leaf buds was performed by Illumina high-throughput sequencing technique, and 59.02 Gb clean data were obtained. A total of 33,612 unigenes were obtained with an N50 value of 1123 bp and transcriptome data of 40,384,342,800 bp by assemblying filtered reads with Trinity software. Then, 3395 DEGs were identified from several different comparisons, and qRT-PCR analysis of 18 randomly selected genes also confirmed same expression levels with transcriptome. These results indicated that our transcriptome data are highly reliable and suitable for downstream analysis.

The flowering initiation is an energy-intensive process in plants, and only occurs when energy reserve is sufficient. Trehalose 6-phosphate (T6P), a key energy reserve, is necessary for normal expression of *FT* gene (Yadav et al. 2014). In *A. thaliana*, sucrose could promote the expression of *TPS* gene, which encodes T6P synthase that catalyzes the production of uridine diphosphate glucose (UDPG) reaction using glucose 6-phosphate (G6P) and T6P. Then, UDPG could activate the expression of *FT* to induce flowering (Wahl et al. 2013). In addition, the mutation of plant vegetative buds into flower buds is caused by the interaction of various hormones, while phytohormones play an important role in flower bud differentiation (Campos et al. 2017). Under certain conditions, plant hormone signals promote or inhibitie the expression of key flowering genes to regulate plant flowering (Conti et al. 2017). In this study, only two KEGG pathways related to flowering were enriched, namely starch and sucrose metabolism pathway and plant hormone signal transduction pathway (Figs. 3, 4 and 5), which indicated that these pathways may be related to light-induced flowering of ginger. Red light and long light treatment could effectively induce differentiation of flower buds in ginger, which also implies that the down-regulated genes involved in theses pathways are possibly negative-regulatory genes related to ginger flowering, while these up-regulated genes play reverse roles.

**Fig. 7:**
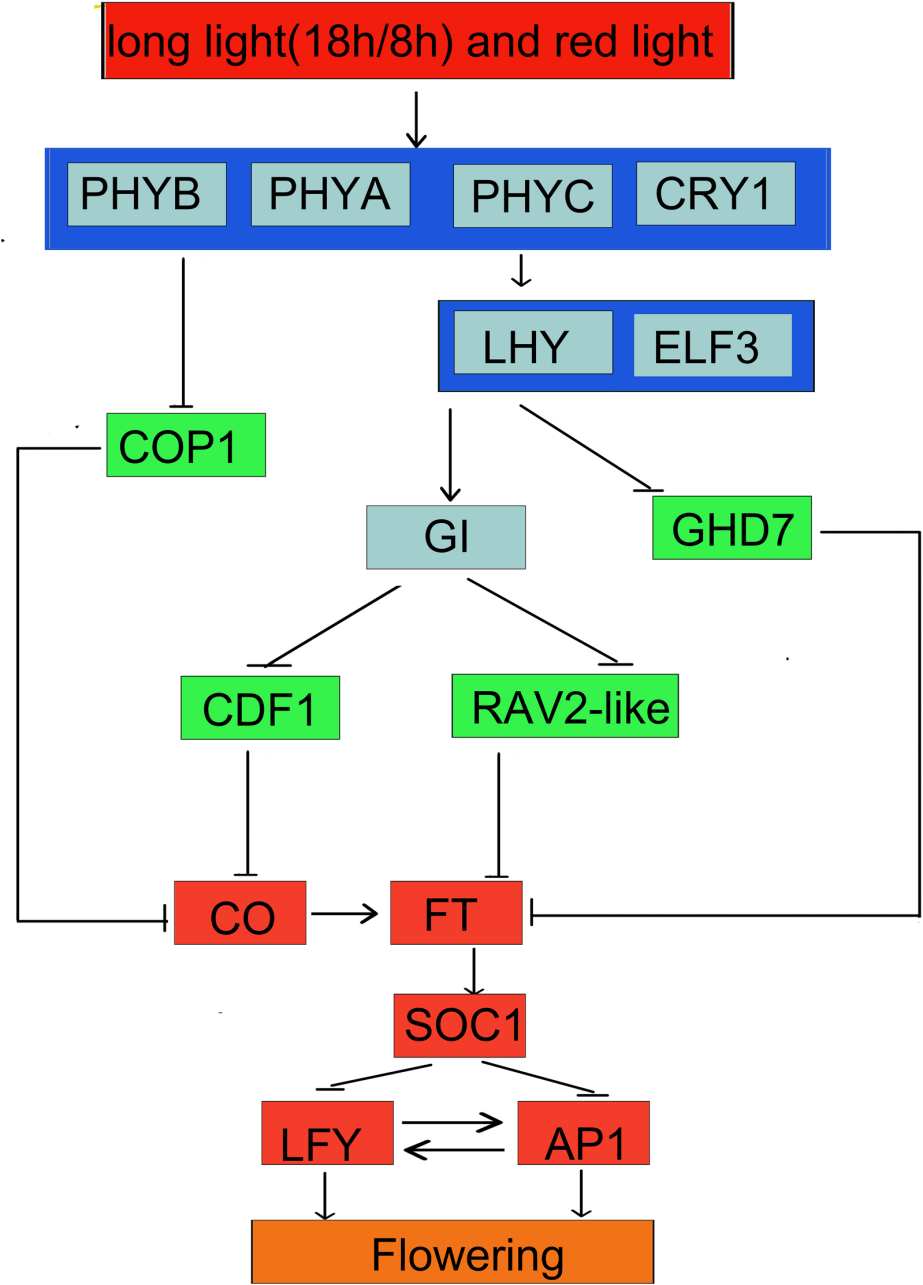
The hypothetical model for ginger flowering regulation network was constructed by reference to *A. thaliana* and *O. sativa* flowering pathway. Change in expression of photoperiod flowering pathway genes after red and long light (18 h light/6 h dark) treatment. Gene names are given within small boxes whose color indicates the change in gene expression (red: upregulated; green: downregulated; blue: no change). Arrows (promotion) and T-bars (repression) indicate regulation

Referring to the flowering regulation network diagram in *A. thaliana* and *O. sativa*, we constructed a hypothetical model for ginger flowering regulation network based on expression patterns of related homologous genes in ginger (Fig. 7). Previous studies have shown that the *RAV2-like* gene can inhibit flowering by inhibiting the expression of *FT* and gibberellin production (Luis et al. 2014). In *A. thaliana*, *AtCDF1* inhibits expression of *CO* and *FT* by binding to promoter of the two genes, resulting in delayed flowering in photoperiodic pathway (Song et al. 2012; Fornara et al. 2009; Imaizumi et al. 2005). *COP1* encodes an ubiquitin E3 ligase, which could promote degradation of CO protein in plants by the ubiquitination process (Liu et al. 2008). In this study, *RAV2-like*, *CFD1*, and *COP1* gene were also identified and were down-regulated expression in FI when compared with LN (Fig. 6). Therefore, *RAV2-like*, *CDF1*, and *COP1* may be important negative regulators in photoperiod flowering pathway of ginger. *GHD7*, a member of *CONSTANS-like* family, inhibits flowering by silencing *Hd3a* (homologous gene of *FT*) in *O. sativa* under long-day condition (Xue et al. 2009). In our study, *GHD7* gene was also identified and the FPKM value decreased by 1456 times in FI vs. LN (Fig. 6). Compared with *RAV2-like*, *CFD1* and *COP1*, the change of FPKM value in *GHD7* is more obvious. Therefore, *GHD7* may be another important negative regulator gene in photoperiod flowering pathway of ginger and may play a leading role. In addition, *PHYA*, *PHYB*, *LHY*, *ELF3* and other genes located in the upstream of the photoperiod pathway were not differentially expressed (Fig. 6), indicating that the down-regulation of *GHD7*, *COP1*, *RAV2-like* and *CDF1* genes may be caused by the comprehensive regulation of the upstream genes of the photoperiod pathway.

*CO* is a key factor that converts light signals into flowering signals, and promotes flowering by activating the expression of *FT* and *SOC1* in *A. thaliana* (Putterill et al. 1995; Samach et al. 2000). *FT* and *SOC1* are floral integrators, which can integrate various flowering pathway signals and promote the expression of *LFY* (Jung et al. 2020). *LFY* play a key role in flower development (Weigel et al. 1992). Mutations in *LFY* can lead to a shift of flower structure to shoots. For example, tomato *LFY* mutants resulted in delayed flowering and greater number of leaves (Molinero-Rosales et al. 1999). *AP1*, another flowering-determining gene, is a MIKC-type MADS-box gene which is located in downstream of *LFY*. It is positively regulated by *LFY*, and the coexpression would promote the transformation of stem meristem into floral meristem (Wagner et al. 1999). In this study, *CO*, *FT*, *SOC1*, *LFY* and two *AP1* genes at different loci (*AP1_1* and *AP1_2*) were identified, and were up-regulated expression in both FI vs. LN and FI vs. LI (Fig. 6). Therefore, *CO*, *FT*, *SOC1*, *LFY* and *AP1* genes may positively regulate ginger flowering downstream of the photoperiod pathway. Besides, *CDF1*, *RAV2-like*, *GHD7* and *COP1* may be the key genes for light-induced flowering of ginger. They are regulated by light, then activate expression of *CO* and *FT* to result in flowering.

## Conclusions

Ginger is an important condiment and medicinal plant, and it is difficult to blossom under natural conditions. However, the flowering related research is important for ginger cross-breeding, which could accelerate the breeding of new varieties. The flowering process is affected by different light conditions and the regulated mechanism of ginger flowering is still unclear. In this study, it was found that 18 h light and red light treatment could effectively induce ginger flowering. In the KEGG pathway, the starch and sucrose metabolism pathway and plant hormone signal transduction pathway may play important roles in ginger flowering. Four down-regulated genes that regulate ginger flowering in the photoperiodic pathway (*GHD7*, *CDF1*, *COP1* and *RAV2-like*) were identified, and they may be important negative regulators for light-induced flowering of ginger. In addition, the significant fold change of FPKM value in *GHD7* indicated that it may play a dominant role in the photoperiod pathway. In conclusion, this study preliminarily revealed the molecular mechanism of ginger flowering induced by photoperiod and light quality, which provided novel insight into ginger breeding.

## Supplementary Information

**Additional file 1: Table S1.** Statistics of functional annotation for ginger transcriptome.

**Additional file 2: Figure S1.** Validation of DEGs by qRT-PCR. **A**, validation of DEGs in LI vs. LN. **B**, validation of DEGs in FI vs. LI. **C**, validation of DEGs in FI vs. LN. (FI: potential flower bud of ginger under induced by photoperiod and light quality; LI: induction treatment of ginger leaf bud; LN: leaf bud of ginger under natural condition)

**Additional file 3: Figure S2.** GO secondary metabolic process of DEGs in FI vs. LI. Asterisks indicate significantly enriched processes. Only 30 terms enriched most significant are shown in this figure. (FI: potential flower bud of ginger under induced by photoperiod and light quality; LI: induction treatment of ginger leaf bud; LN: leaf bud of ginger under natural condition)

**Additional file 4: Figure S3.** GO secondary metabolic process of DEGs in LI vs. LN. Asterisks indicate significantly enriched processes. Only 30 terms enriched most significant are shown in this figure. (FI: potential flower bud of ginger under induced by photoperiod and light quality; LI: induction treatment of ginger leaf bud; LN: leaf bud of ginger under natural condition)

**Additional file 5: Figure S4.** GO secondary metabolic process of DEGs in FI vs. LN. Asterisks indicate significantly enriched processes. Only 30 terms enriched most significant are shown in this figure. (FI: potential flower bud of ginger under induced by photoperiod and light quality; LI: induction treatment of ginger leaf bud; LN: leaf bud of ginger under natural condition)

**Additional file 6: Table S2.** Annotation information of differentially expressed genes.

## Data Availability

The datasets used and/or analysed during the current study are available from the corresponding author on reasonable request.

## Abbreviations

FI: Potential flower bud of ginger under induced by photoperiod and light quality
LI: Induction treatment of ginger leaf bud
LN: Leaf bud of ginger under natural condition
NR: Non-redundant protein sequences in NCBI
GO: Gene ontology
KEGG: Kyoto encyclopedia of genes and genomes
STRING: Search tool for the retrieval of interacting genes/proteins
DEGs: Differentially expressed unigenes
qRT-PCR: Real-time quantitative PCR
FPKM: Fragments per kilobase of transcript per million mapped reads
T6P: Trehalose 6-phosphate
UDPG: Uridine diphosphate glucose
G6P: Glucose 6-phosphate
