# Supplementary material for: Transcriptome profiles reveal gene regulation of ginger flowering induced by photoperiod and light quality
Source: Bot Stud. 2023 May 27;64:12. doi: 10.1186/s40529-023-00388-7 (PMC10219913; doi:10.1186/s40529-023-00388-7)
Supplement: Supplementary file 1 — Additional file 1: Table S1. Statistics of functional annotation for ginger transcriptome. [file 40529_2023_388_MOESM1_ESM.docx]

| **Table S1** Statistics of functional annotation for the ginger transcriptome | | | | | |
| --- | --- | --- | --- | --- | --- |
| NR KEGG GO STRING Total | | | | | |
| Number | 22,123 | 23,624 | 22,538 | 29,111 | 29,985 |
| Ratio | 65.81% | 70.28% | 67.05% | 86.61% | 88.91% |
