# Supplementary figures and images for: Transcriptome profiles reveal gene regulation of ginger flowering induced by photoperiod and light quality
Source: Bot Stud. 2023 May 27;64:12. doi: 10.1186/s40529-023-00388-7 (PMC10219913; doi:10.1186/s40529-023-00388-7)

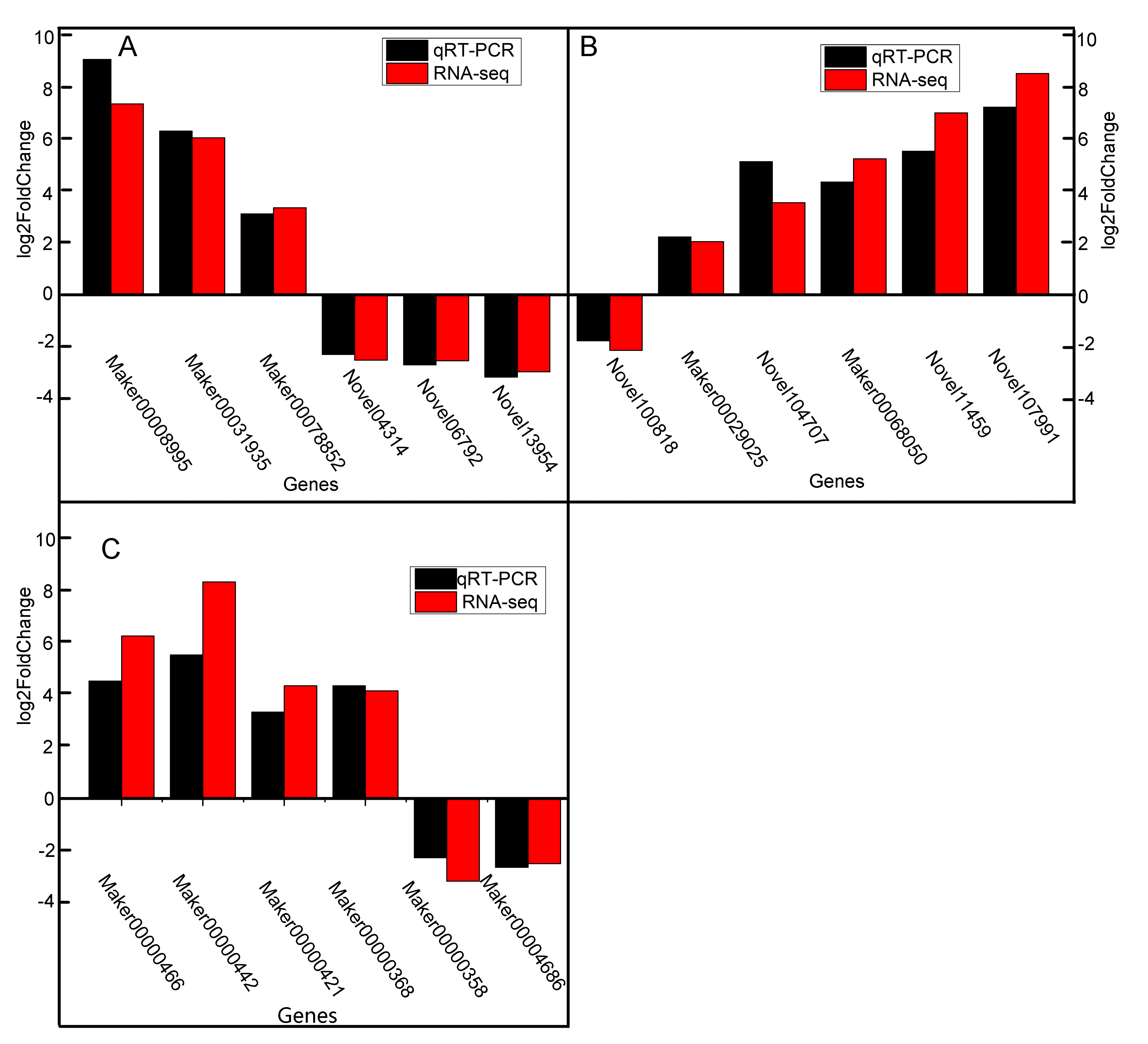

Supplement: Supplementary file 2 — Additional file 2: Figure S1. Validation of DEGs by qRT-PCR. A, validation of DEGs in LI vs. LN. B, validation of DEGs in FI vs. LI. C, validation of DEGs in FI vs. LN. (FI: potential flower bud of ginger under induced by photoperiod and light quality; LI: induction treatment of ginger leaf bud; LN: leaf bud of ginger under natural condition) [file 40529_2023_388_MOESM2_ESM.jpg]

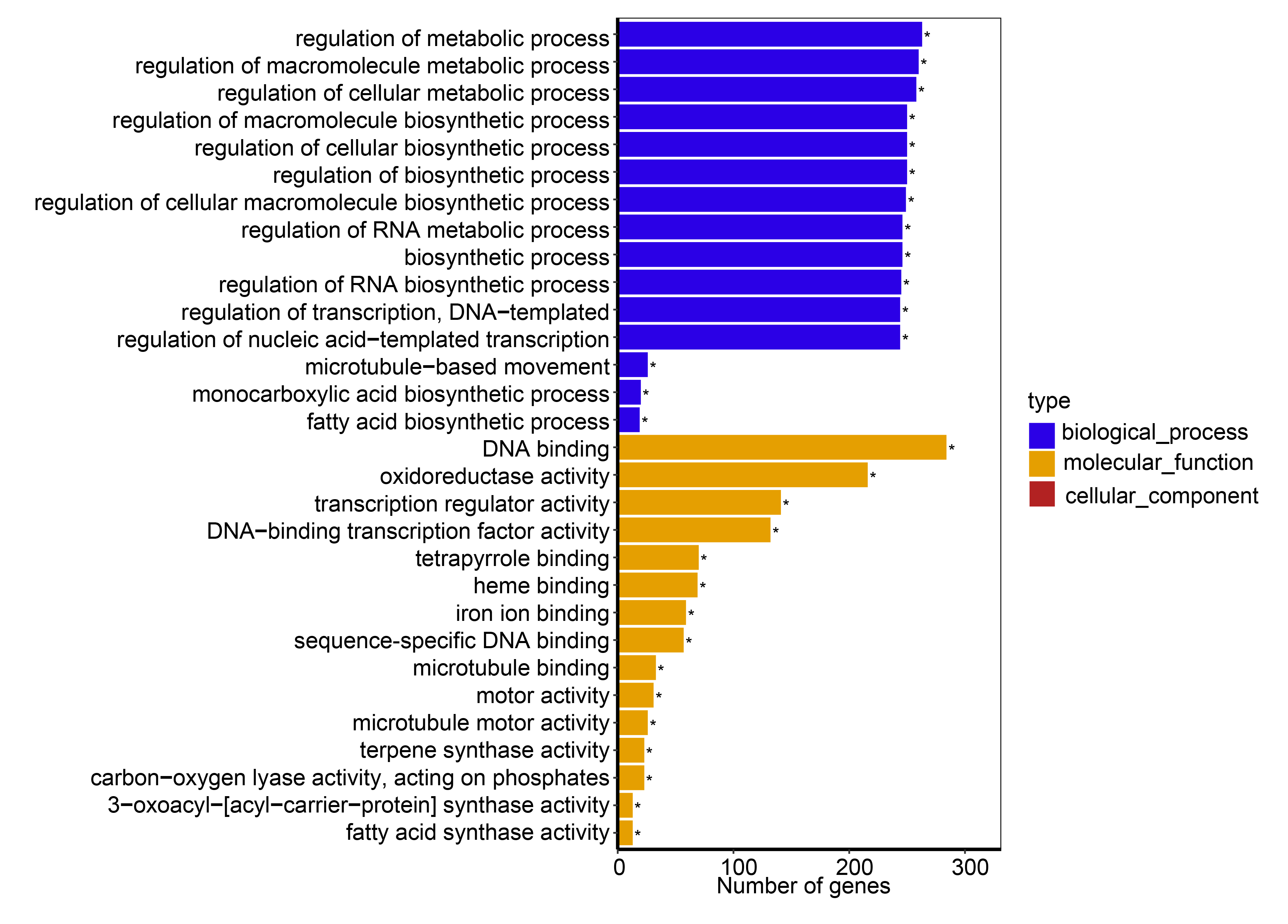

Supplement: Supplementary file 3 — Additional file 3: Figure S2. GO secondary metabolic process of DEGs in FI vs. LI. Asterisks indicate significantly enriched processes. Only 30 terms enriched most significant are shown in this figure. (FI: potential flower bud of ginger under induced by photoperiod and light quality; LI: induction treatment of ginger leaf bud; LN: leaf bud of ginger under natural condition) [file 40529_2023_388_MOESM3_ESM.jpg]

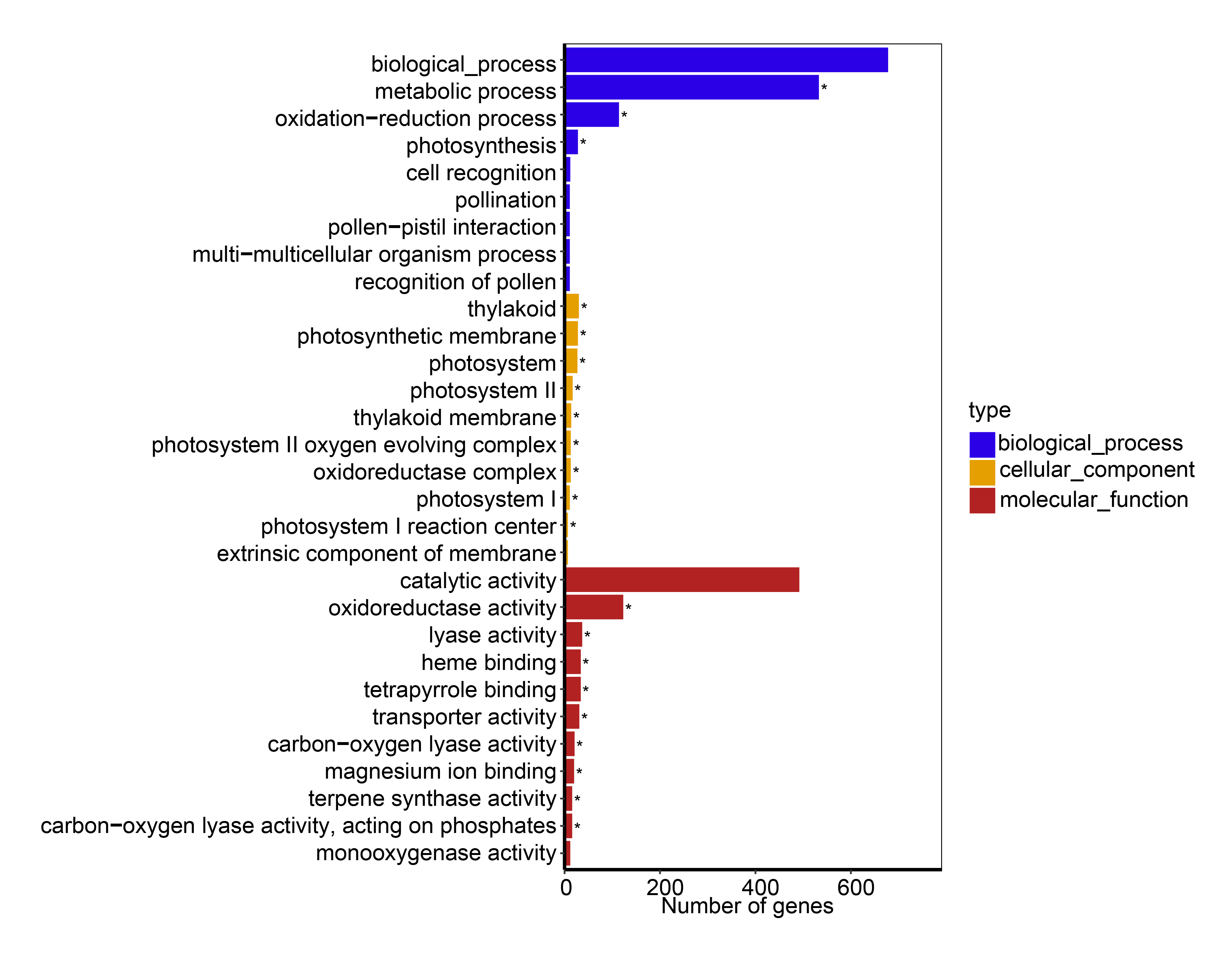

Supplement: Supplementary file 4 — Additional file 4: Figure S3. GO secondary metabolic process of DEGs in LI vs. LN. Asterisks indicate significantly enriched processes. Only 30 terms enriched most significant are shown in this figure. (FI: potential flower bud of ginger under induced by photoperiod and light quality; LI: induction treatment of ginger leaf bud; LN: leaf bud of ginger under natural condition) [file 40529_2023_388_MOESM4_ESM.jpg]

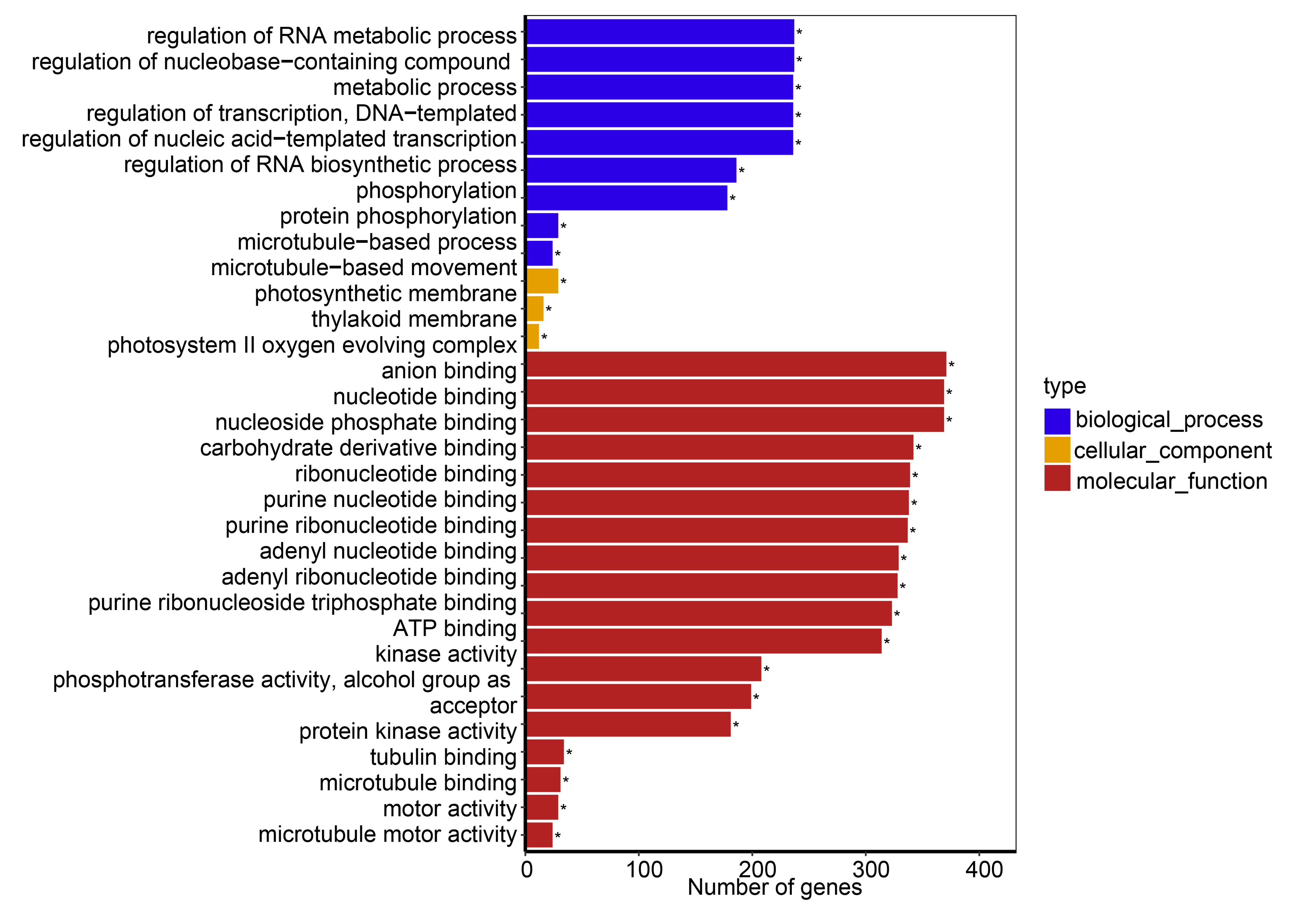

Supplement: Supplementary file 5 — Additional file 5: Figure S4. GO secondary metabolic process of DEGs in FI vs. LN. Asterisks indicate significantly enriched processes. Only 30 terms enriched most significant are shown in this figure. (FI: potential flower bud of ginger under induced by photoperiod and light quality; LI: induction treatment of ginger leaf bud; LN: leaf bud of ginger under natural condition) [file 40529_2023_388_MOESM5_ESM.jpg]
