# Supplementary material for: Transcriptome profiles reveal gene regulation of ginger flowering induced by photoperiod and light quality
Source: Bot Stud. 2023 May 27;64:12. doi: 10.1186/s40529-023-00388-7 (PMC10219913; doi:10.1186/s40529-023-00388-7)
Supplement: Supplementary file 6 — Additional file 6: Table S2. Annotation information of differentially expressed genes. [file 40529_2023_388_MOESM6_ESM.docx]

| **Table S2** Annotation information of differentially expressed genes | | |
| --- | --- | --- |
| Gene name | Gene ID | Gene annotation |
| CDF1 | Maker00068294 | cyclic dof factor 1-like |
| CO  FT | Novel01171  Maker00069437 | CONSTANS  flowering locus T |
| AP1*_*1  AP1*_*2  SOC1 | Novel01801  Novel09969  Maker00030449 | APETALA1-like protein  APETALA1-like protein  MADS-box protein SOC1-like |
| GHD7 | Novel02361 | transcription factor GHD7-like |
| COP1 | Novel00246 | hypothetical protein SHCRBa_004_O05_R_50 |
| RAV2-like | Novel07461 | AP2/ERF and B3 domain-containing transcription repressor RAV2-like |
| LFY  PHYA_1  PHYA_2  PHYA_3  PHYB  PHYC  CRY1_1  CRY1_2  CRY1_3  CRY1_4  LHY  ELF3_1  ELF3_2  ELF3_3  ELF3_4  GI_1  GI_2 | Novel10831  Maker00058073  Novel14594  Novel06537  Novel14567  Maker00060967  Maker00014478  Maker00029304  Maker00060884  Novel09129  Novel12865  Maker00000625  Maker00026429  Maker00030674  Maker00070568  Maker00016163  Maker00069072 | F-box only protein 13  phytochrome A-associated F-box protein  phytochrome A-associated F-box protein  phytochrome A  phytochrome B  phytochrome C  cryptochrome-1  cryptochrome-1  cryptochrome-1  cryptochrome-1  Homeodomain-like superfamily protein  hydroxyproline-rich glycoprotein family protein, partial  hydroxyproline-rich glycoprotein family protein, partial  hydroxyproline-rich glycoprotein family protein, partial  hydroxyproline-rich glycoprotein family protein, partial  protein GIGANTEA isoform X2  protein GIGANTEA |
